# Analysis of Thermal Sensitivity of Human Cytomegalovirus Assayed in the Conventional Conditions of a Human Milk Bank

**DOI:** 10.3389/fped.2021.640638

**Published:** 2021-07-27

**Authors:** Antoni Gayà, Massimo Rittà, David Lembo, Paola Tonetto, Francesco Cresi, Stefano Sottemano, Enrico Bertino, Guido E. Moro, Javier Calvo, Manuela Donalisio

**Affiliations:** ^1^Banc de Teixits, Fundació Banc de Sang i Teixits de les Illes Balears (FBSTIB), Palma, Spain; ^2^Cell Therapy and Tissue Engineering Group (TERCIT), Balearic Islands Health Research Institute (IdISBa), Palma, Spain; ^3^Laboratory of Molecular Virology and Antiviral Research, Department of Clinical and Biological Sciences, University of Turin, Turin, Italy; ^4^Neonatal Care Unit of the University, City of Health and Science Hospital, Turin, Italy; ^5^Italian Association of Human Milk Banks, Milan, Italy

**Keywords:** holder pasteurization, donor human milk, viral inactivation, human milk bank, breast milk, cytomegalovirus

## Abstract

One of the main concerns in human milk banks (HMB) is the transmission of human cytomegalovirus (HCMV) that could be present in the milk of infected women. There are consistent data showing that this virus is destroyed by Holder pasteurization (62.5°C for 30 min), but there is a lack of information about the response of the virus to the treatment at lower temperatures in strict HMB conditions. In order to analyze the effectiveness of different temperatures of pasteurization to eliminate HCMV in human milk, a preliminary assay was performed incubating HCMV-spiked raw milk samples from donor mothers at tested temperatures in a PCR thermocycler and the viral infectivity was assayed on cell cultures. No signs of viral replication were observed after treatments at temperatures equal or >53°C for 30, 20, and 10 min, 58°C for 5 min, 59°C for 2 min, and 60°C for 1 min. These data were confirmed in a pasteurizer-like model introducing HCMV-spiked milk in disposable baby bottles. No viral infectivity was detected on cell cultures after heating treatment of milk for 30 min at temperatures from 56 to 60°C. Thus, our results show that by using conventional pasteurization conditions, temperatures in the range of 56–60°C are enough to inactivate HCMV. Consequently, we consider that, in order to provide a higher quality product, the current recommendation to pasteurize both mother's own milk and donated milk at 62.5°C must be re-evaluated.

## Introduction

It is widely accepted that breast milk is the optimal source of nutrition for infants (1, 2). However, it has been reported that only 27% of mothers were able to provide sufficient milk to their preterm infants (3). In this situation, the preferred alternative is donated human milk, and only if this is not available, preterm formula (2).

As any other Substance of Human Origin (SOHO), human milk has a potential risk of infectious disease transmission. In order to minimize this risk, several strategies are applied (4), including the thermal treatment of donated milk. This treatment is known as pasteurization and is defined as the process of heating a food, usually a liquid, at a specific temperature during a predefined period of time and then immediately cooling it after it is removed from the heat.

Human milk is a complex mixture of essential nutrients and bioactive molecules designed not only to cover the nutritional needs of the neonate but also to facilitate the process of maturation of various organs as gut and brain (5). From a nutritional standpoint, this heat-treatment does not seem to affect the macronutrient composition (protein, carbohydrates and lipids, including polyunsaturated fatty acids) of milk. However, there are many evidences showing that there is a drastic decrease in lipase activity, as well as a substantial drop in the concentrations of various biological factors such as IgA, lactoferrin, lysozyme, cytokines and growth factors (6, 7). Thus, in the process of securing human milk, there is a decrease of milk quality due to the destruction of some biological components. The literature shows the enormous importance that the biological factors present in milk represent for the development and maturation of the newborn (8, 9). That is why one of the goals of milk banks must be to process human milk trying to damage these factors as little as possible during processing.

Although the trend toward a better preservation of biological factors is focused through the development of new treatments (10, 11), such as High Temperature Short Time (HTST) (12, 13), High pressure processing (HPP) (14) or Ultraviolet (UV) irradiation (15), there is a difficulty to transfer into practice, given the lack of appropriately scaled and economically affordable equipment to be used in milk banks. A simpler and alternative approach would be to optimize the conventional pasteurization technique in such a way that, maintaining the destructive capacity on infectious elements, it retains as much as possible the biological components of human milk. This option has the advantage that it would allow to reuse the pasteurization equipment currently available in most milk banks, without needing to acquire new equipment, simply by adjusting the temperature.

There are clear evidences that temperatures lower than those recommended nowadays (Holder pasteurization; 30 min at 62.5°C) are less deleterious with the biological components of milk, whereas, retaining the capacity to destroy bacterial and viral [HIV, HTLV-I, Polioviruses, Chikungunya Virus (CHIKV), West Nile Virus (WNV)] contaminations in donor human milk (16, 17). Unfortunately, there are few data about the sensitivity of Human Cytomegalovirus (HCMV) to the range of temperatures between 56 and 63°C. In a recent paper (18) Maschmann et al., concluded that temperatures above 60°C for performing short-term pasteurization are enough for the purpose of CMV inactivation. However, they used a short-term heating protocol done with a prototype of a commercially available machine (Virex, Klaus Lauf, Germany) and aliquots of 20 ml of breast milk, instead of the conventional equipment (pasteurizer) and the conventional volumes (50–250 ml) of a human milk bank.

Therefore, the aim of this work has been to evaluate the temperature sensitivity of HCMV during the processing of donor breast milk under the same conditions that are commonly used in human milk banks. If it is confirmed that HCMV is destroyed at temperatures lower than that used in Holder pasteurization, as shown in the literature for most infectious agents (16, 17), the possibility of a thermal treatment of donated milk more respectful with its biological components would be possible and this would be beneficial for the receptors.

## Materials and Methods

### Milk Samples

Human milk samples were obtained from the Human Milk Bank of the Città della Salute e della Scienza of Turin, Italy, in 2018. The study has been reviewed and approved by the Ethics Committee of Italian Association of Human Milk Banks (Milan, Italy) 1 month before the beginning. The donors cleaned their hands and breasts according to the Italian HMB guidelines. The milk specimens were collected in sterile bisphenol-free polypropylene bottles using a breast pump and stored at −20°C until processed. The specimens were thawed overnight in refrigerated conditions and then processed according to the appropriate technique.

### Cells

Human Foreskin Fibroblasts (HFF-1) (ATCC SCRC-1041) at low-passage-number (<30) were grown as monolayers in Dulbecco's Modified Eagle Medium (DMEM) (Sigma, St. Louis, MO, USA) supplemented with 10% fetal calf serum (FCS) (Sigma), 1 mM sodium pyruvate and 1% antibiotic-antimycotic solution (Zell Shield, Minerva Biolabs, Berlin, Germany).

### Virus

A (BAC)-derived HCMV strain Towne, incorporating the GFP sequence was propagated on HFF-1s. The GFP marker facilitates identification of infected eukaryotic cells (19). Human cytomegalovirus strain AD169 was purchased from the ATCC (VR-538). Viral stocks were prepared by infecting HFF-1s at a virus-to-cell ratio of 0.01. The cells were incubated in DMEM supplemented with 2% heat-inactivated FCS and cultured until a marked cytopathic effect was observed. Stocks were centrifugally clarified and frozen at −80°C. Virus titers were determined by fluorescent focus assay for strain Towne and HCMV AD169 titers were determined on HFF-1 cells using the median tissue culture infective dose (TCID50) method.

### Virus Spiking Experiments

Raw milk samples from the mothers were spiked with an inoculum of HCMV strain Towne of 50,000 foci forming units (FFU) in 100 μl of milk for PCR-thermocycler experiments and in 1 ml of milk for “disposable baby bottles” experiments. Two inocula of 10^4^ and 3 × 10^5^ TCID50 were used in 1 ml of milk for “disposable baby bottles” experiments for HCMV strain AD169. Final volumes of milk were 100 μl for pasteurization experiments performed in a PCR-thermocycler and 130 ml in baby bottles incubated in water bath.

### Pasteurization

Preliminary experiments were performed in a PCR-thermocycler (C1000 Thermal Cycler, Bio-Rad). In a first study, each 100 μl of HCMV-Towne-spiked milk specimens were incubated for 30 min at different temperatures (48–63°C) and then cooled at 4°C. An additional sample of spiked milk was incubated for 30 min at 4°C as a control. The virus-milk mixtures were then diluted in cell culture medium (1:2) and added to pre-plated cells to test virus infectivity, as described below. In a second study, a time-kinetics of heat treatment was carried out for 30, 20, 10, 5, 2, and 1 min at different temperatures (48–63°C).

Then, pasteurization experiments were performed in disposable baby bottles incubated in a water bath, a procedure resembling common pasteurization practice currently in use in human donor milk banks for milk pasteurization. In this case, 1 ml HCMV-spiked milk in dialysis membrane tubing was inserted in disposable milk storage baby bottles containing 130 ml of milk. For each experiment, a control included 1 ml of milk challenged with constant amount of HCMV and kept at 4°C for 30 min. All experiments were performed in duplicate. After thermal treatment, the residual HCMV-challenged milk was collected, and the HCMV infectivity assay was performed as described below.

### HCMV Infectivity Assay

HFF-1 cells were seeded in 96-well plates at a density of 5 × 10^3^ cells/well and incubated at 37°C in a 5% CO_2_ atmosphere for 24 h. Cells were infected with a 1:2 dilution of the pasteurized spiked milk in DMEM supplemented with 2% of heat-inactivated FBS for 2 h at 37°C. Then monolayers were washed five times and incubated with 1.2%-methylcellulose DMEM medium with 2% FBS. HCMV-Towne-infected cells were visualized as green fibroblasts on a confocal fluorescence microscope (LSM510, Carl Zeiss, Germany) and counted 5 days post-infection. After 5 or 10 days of incubation, HCMV-AD169-infected cells were fixed and subjected to HCMV specific immunostaining using an anti-HCMV IEA monoclonal antibody (11-003; Argene, France) and an UltraTech HRP streptavidin–biotin detection system (Beckman Coulter, France), in order to detect the viral IEA protein expression (20). All the experiments were performed in triplicate.

### Cell Viability Assay

Cell viability was assessed using the 3-(4,5-dimethylthiazol- 2-yl)-5-(3-carboxymethoxyphenyl)-2-(4-sulfophenyl)-2H-tetrazolium assay as described previously (21). The effect of breast milk dilutions on cell viability was expressed as a percentage of absorbance values of treated cells compared with those of cells incubated with culture medium alone. The 50%-cytotoxic dilutions (CD50) and 95% confidence intervals were determined with Prism 4 software (GraphPad Software, USA).

## Results and Discussion

### Effect of Different Time-Temperature Combinations on HCMV Infectivity

To determine the effectiveness of different time-temperature combinations of pasteurization on HCMV infectivity in human milk, raw milk samples from donor mothers were spiked with an inoculum of GFP-expressing HCMV-Towne. The inoculated milk was then heat-treated in the range 48–63°C for 1, 2, 5, 10, 20, and 30 min in a PCR-thermocycler or kept at 4°C. Diluted samples were then analyzed by HCMV infectivity assays on inoculated cell monolayers and examined for GFP expression by fluorescence microscopy, as described in Supplementary Figure 1A.

The time-kinetics study of HCMV inactivation at different degrees revealed that no signs of HCMV replication were observed in treatments at temperatures ≥60°C for 1 min, at 59°C for 2 min, in the range from 54 to 58°C for 5 min, and at 53°C for 10 min (Supplementary Figure 1B). By contrast, the exposition to 52, 51, 50, 49, and 48°C resulted in a temperature-dependent reduction of infectivity without the total elimination of infected cells in the considered range of time.

Supplementary Figure 1C shows that a high number of GFP-expressing cells are visible in monolayers infected with only viruses maintained at 4°C for 30 min as positive control (control virus). Instead, a reduced number of green cells was reported when virus was incubated with milk at 4°C for 30 min (control milk plus virus) due to the antiviral properties of human breast milk, as we published recently (22). By contrast, the viral infectivity was completely abolished, and no GFP-expressing cells could be detected in cell monolayers infected with pasteurized HCMV-spiked milk at temperatures and times before mentioned (Milk plus virus). Preliminary experiments were conducted to determine whether the 1:2 dilution of the pasteurized spiked milk, used in the viral assays, was toxic on cells. No impact of the diluted milk sample on cell viability was observed (data not shown).

According to the literature, long time pasteurizations for 30 min at temperatures lower than 62.5°C were effective to inhibit most of viruses. In fact, CHIKV and WNV are inactivated at 58°C for 30 min, Polioviruses at 55°C for 30 min, HTLV-I at 56°C for 30 min, HCV at 60°C for 30 min and HIV at 60°C at a time <30 min (23–27). Indeed, 30 min can be considered the optimal time for a broad inactivation of breast milk-transmitted viruses. For these reasons, further studies of pasteurization were performed at this time.

### Investigation of the Effect of Different Temperatures on HCMV Replication in a Pasteurizer-Like Model

In order to investigate the effect of different pasteurization temperatures on HCMV infectivity in a pasteurizer-like model, a constant amount of HCMV strain-Towne was challenged in 1 ml milk in dialysis membrane bags inserted in disposable milk storage baby bottles containing 130 ml of milk. The bottles underwent heating treatment for 30 min at 56, 57, 58, 59, and 60°C and then, the whole spiked milk, recovered from the dialysis membrane bags, was inoculated on HFF-1 cell monolayers for the detection of viral infectivity examining the GFP expression as previously described (Supplementary Figure 2A). The thermal profiles of pasteurizations are reported in Supplementary Figure 3. As reported in Supplementary Figures 2B,C, HCMV was inactivated by any tested time-temperature combination, whereas, all the wells of controls exhibited clear signs of HCMV infection. The average number of FFU/ml was 2050 in the control milk plus virus (at 4°C for 30 min), and uncountable for the control virus (at 4°C for 30 min). These data confirm that the thermal treatments at 60, 59, 58, 57, and 56°C for 30 min inactivated HCMV completely in our model system.

**Figure 1:**
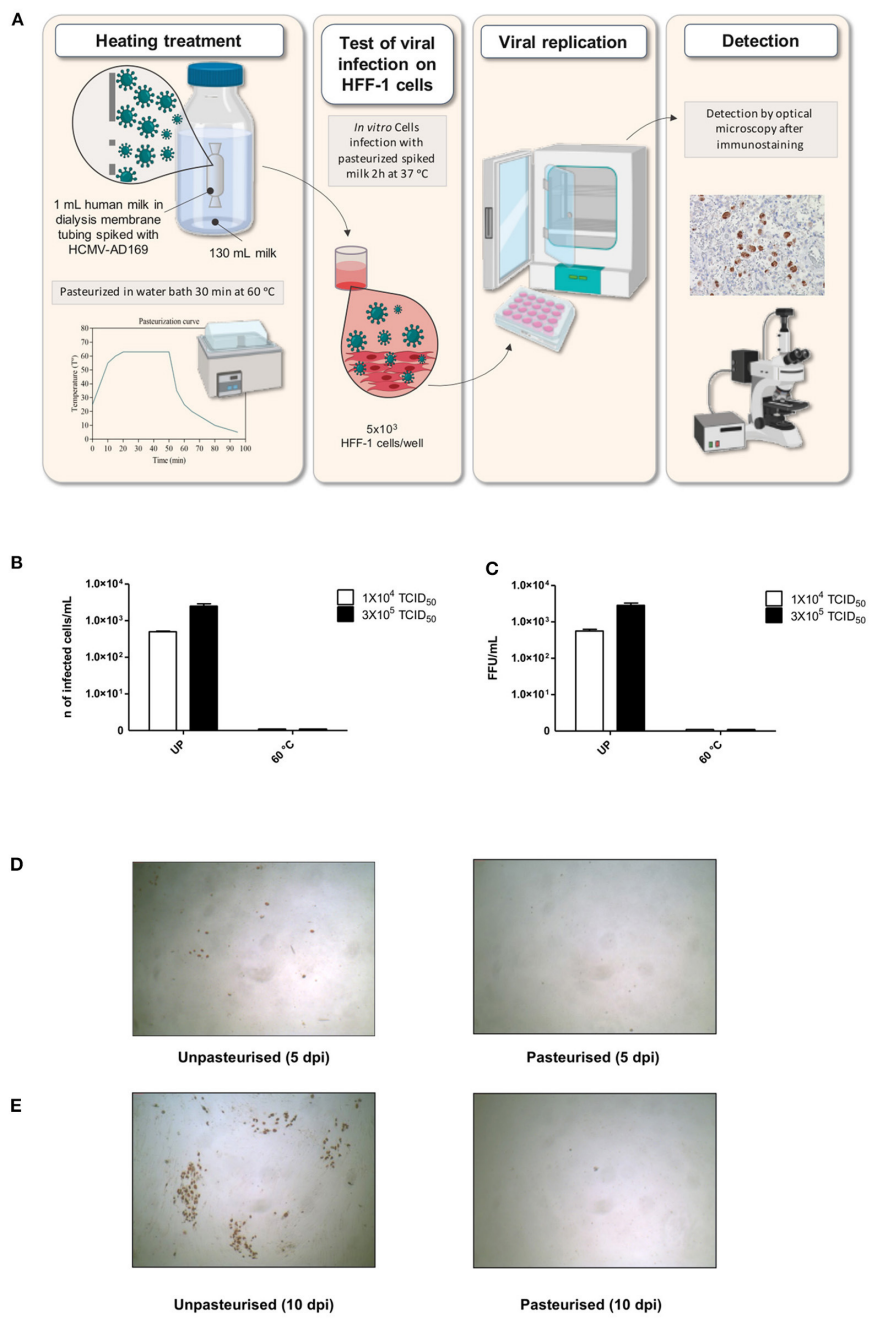
Effects of treatment at 60°C for 30 min on HCMV strain AD169 infectivity at two different titers in a pasteurizer-like model. **(A)** Heat treatment and infection protocol for HCMV. **(B,C)** In the graphs, HCMV infectivity of heat-treated milk samples spiked with HCMV AD169 at two titers (1 × 10^4^ TCID50 and 3 × 10^5^ TCID50) is reported as number of infected cells/ml at 5 days post infection **(B)** and as FFU/ml at 10 days post infection **(C)**. **(D,E)**. Representative figures of HFF-1 cells treated with milk samples spiked with HCMV (3 × 10^5^ TCID50) and treated at 4°C for 30 min (unpasteurised, left) or at 60°C for 30 min (pasteurized, right) at 5 days post infection **(D)** and 10 days post infection **(E)**. Infected cells and foci are brown by immunostaining. UP, unpasteurised.

To validate our results at 60°C, the assay was performed in the pasteurizer-like model with a different laboratory HCMV strain, AD169, used as model in pasteurized HCMV inactivation assays (28). Spiking of the breast milk samples was done by adding two viral inocula (10^4^ and 3 × 10^5^ TCID50 per milliliter of milk) and the viral infectivity was assessed by an indirect immunoperoxidase staining procedure at 5 and 10 days post infection (dpi) (Figure 1A). At 5 dpi, the read-out of the viral infectivity was the number of infected cells or foci, brown stained by the immediate early antigen (IEA) staining/ per milliliter milk (Figure 1D). As reported in Figure 1B, a total inhibition of viral infectivity was observed at 60°C for 30 min at 5 days post infection for both viral inocula. The concentration of virus in the different inocula is much higher than what is detected in any wild type infected breast milk (18). At 10 dpi, brown foci or plaques, generated by the cell-to-cell viral spread, were detected in the control, and again a total absence of detectable cytopathic effect was obtained at 60°C for 30 min (Figures 1C,E).

Today the Holder pasteurization (30 min at 62.5°C) is still considered as “gold standard” to avoid transmission of pathogens to breastfed infants. However, its harmful effects on nutritional and immunological components of breast milk have induced the study of different ways of HCMV inactivation (10). Bapistella et al. (29) assessed the effectiveness of short-term pasteurization (62°C for 5 s) in preventing HCMV transmission *via* breast milk observing a reduction in the proportion of HCMV transmission from 20.5 to 2.3% preterm infants. Recently, HTST pasteurization has been reported to be a valuable alternative technology to increase the retention of some biological features, like the antiviral properties of human milk (30). Klotz et al. (28) evaluated the antiviral and antibacterial efficacy of different time-temperature combinations of HTST observing that the treatment inactivated HCMV but is less effective than Holder pasteurization in bacterial count reduction. A new short-term heat treatment subjecting samples for 5 s to different temperatures (55–72°C) has been recently studied for its impact on HCMV inactivation (18). However, this is a very small-scale technique, not useful to be used in conventional HMB to process liters of human milk.

Herein, we evaluated preliminarily the impact of different temperature-time combinations on HCMV infectivity in a PCR-thermocycler. Analyzing the exposition of virus-spiked milk between 48 and 63°C, our experiments showed that the rate of HCMV inactivation was 100% at temperatures equal or >60°C for 1 min, whereas, lower temperatures gradually diminished the efficacy and required a longer time to totally inhibit the infectivity. Our experiments in a pasteurizer-like model confirmed the complete HCMV inactivation treating virus-spiked milk for 30 min in the range from 56 to 60°C and validated the efficacy of the pasteurization at 60°C for 30 min against two laboratory HCMV strains.

As regards thermo-sensitivity in bacteria, few studies have focused on the susceptibility of the microbial strains. The most complete study addressing this issue was published by Czank et al. (31). They observed that incubating human milk, spiked with different bacterial species, at 57°C for 30 min was enough to reduce all bacterial species tested by 99.9%. The authors conclude that the current practice, Holder pasteurization, may be considered as excessive for pasteurizing human milk donated to the milk banks. This overheating is probably explained, as we discussed in a previous paper (16), because the temperature chosen to pasteurize in the human milk banks, 62.5°C for 30 min, comes from data obtained with cow's milk in dairy industry and not from a systematic analysis using human milk. It should be noted that the analysis was done by using an experimental pasteurizer, designed specifically for that study and not in the conventional conditions of a human milk bank. Working in these conventional conditions, we have obtained preliminary data (Calvo et al., manuscript in preparation) confirming that, by pasteurizing at 60°C, the flora usually present in the milk samples received at our milk bank is completely destroyed. The exception is *Bacillus spp*, that, as it is well-known, has the ability to resist pasteurization due to its sporulation capacity (32). In fact, *Bacillus cereus* has been described as the primary cause of rejection for pasteurized human milk (33, 34).

We consider that our results are especially relevant in order to protect the biological factors present in human milk, as it has been described by Czank et al. (31) that the temperature and not the holding time, is the critical element for the retention of IgA, lysozyme and lactoferrin, with significant differences in the retention of these three proteins by modifying the temperature of treatment just by 1°C. These data, however, were obtained by using a specially designed pasteurizer, different than those commonly used in human milk banks. For that reason, we have started a project to analyze the effect of different temperatures on some biological factors, after the heat treatment of human milk using the conventional conditions used in human milk banks.

In conclusion, our data clearly confirm that the heat treatment for 30 min at 60°C guarantees the total inactivation of HCMV. Taking into account that previous reports suggest that this is also applicable to other breastfeeding transmitted viruses, like HIV, HTLV and Parvovirus B19 (16, 17), we consider that this variant of Holder pasteurization may be a good compromise between the microbial inactivation and the preservation of nutritional properties using the same pasteurizer equipment found in most of Human Milk Banks. These aspects make the procedure of pasteurization favorable especially in low-income countries. A limitation of this study is to have performed experiments on artificially spiked breast milk samples, infected with laboratory HCMV. Further studies will be necessary to confirm the inactivation of infectivity of free clinical strains and cell associated ones in naturally CMV-infected breast milk at 60°C for 30 min. Moreover, future studies are required in relation with the bacterial destruction and the preservation of the biological components to definitively validate this variant of pasteurization.

## Data Availability Statement

The raw data supporting the conclusions of this article will be made available by the authors, without undue reservation.

## Ethics Statement

The studies involving human participants were reviewed and approved by Ethics Committee of Italian Association of Human Milk Banks (Milan, Italy). The patients/participants provided their written informed consent to participate in this study.

## Author Contributions

JC, AG, and MD conceived and designed the study and wrote the first draft of the manuscript. PT and SS collected the milk samples. MR performed the investigations. GM, DL, EB, and FC reviewed and edited the manuscript. All authors contributed to manuscript revision, read, and approved the submitted version.

## Conflict of Interest

The authors declare that the research was conducted in the absence of any commercial or financial relationships that could be construed as a potential conflict of interest.

## Publisher's Note

All claims expressed in this article are solely those of the authors and do not necessarily represent those of their affiliated organizations, or those of the publisher, the editors and the reviewers. Any product that may be evaluated in this article, or claim that may be made by its manufacturer, is not guaranteed or endorsed by the publisher.
