# Supplementary material for: Analysis of Thermal Sensitivity of Human Cytomegalovirus Assayed in the Conventional Conditions of a Human Milk Bank
Source: Front Pediatr. 2021 Jul 27;9:640638. doi: 10.3389/fped.2021.640638 (PMC8353116; doi:10.3389/fped.2021.640638)
Supplement: Supplementary file 1 [file Data_Sheet_1.docx]

**Supplementary Figure 1.** **Effects of temperature at different times on HCMV strain Towne infectivity.** Panel A. Heat treatment and infection protocol for HCMV. Panel B. On the Y-axis, the length of time at which HCMV infectivity was inhibited completely is reported for each temperature investigated (48-63 °C). Panel C. Representative figures of HFF-1 cells infected with HCMV (4 °C for 30 minutes, control virus, left), HCMV-spiked milk samples treated at 4 °C for 30 minutes (control milk plus virus, middle), and HCMV-spiked milk sample treated at 53 °C for 10 minutes (milk plus virus, right). In green, fluorescent HCMV foci.


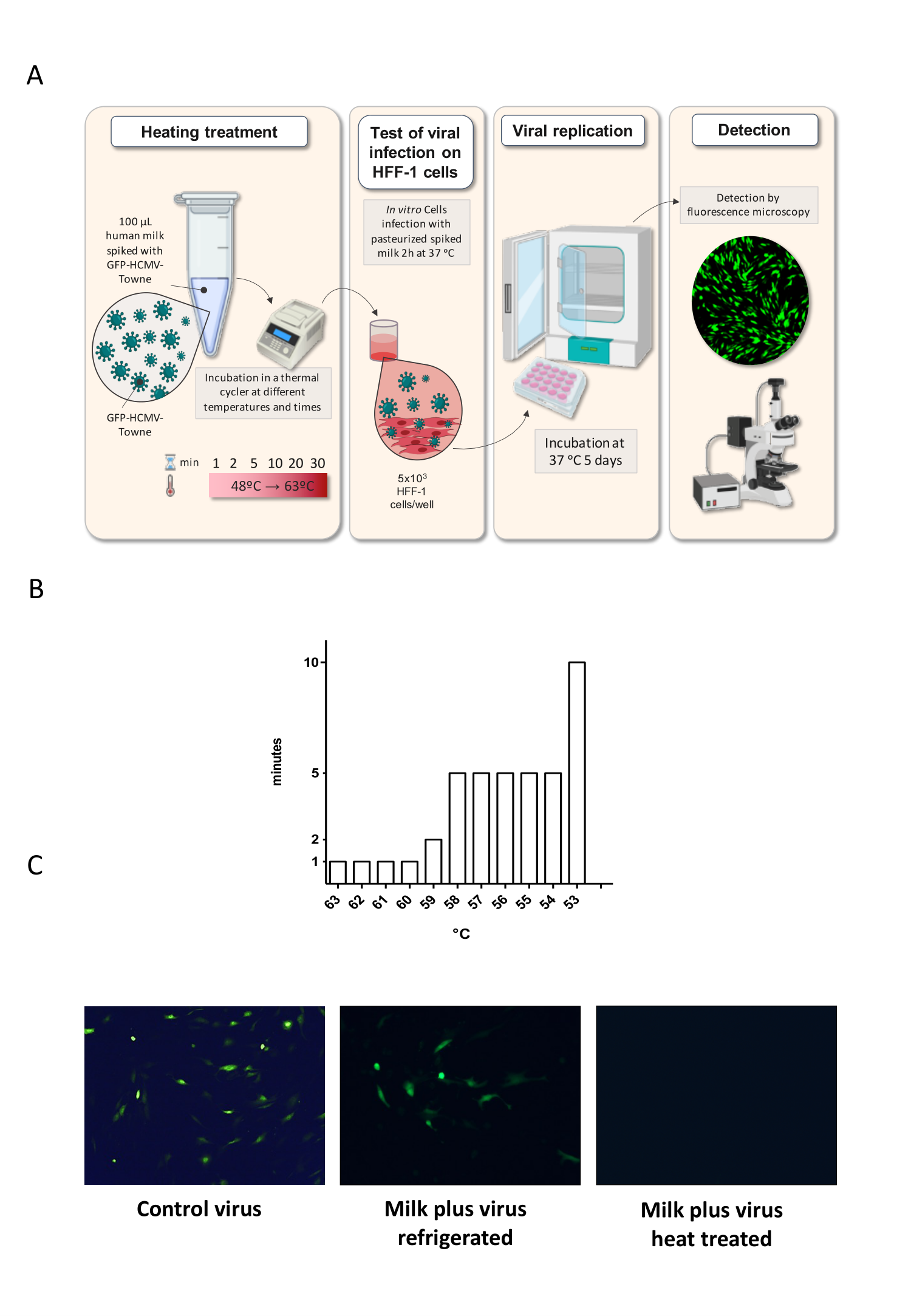


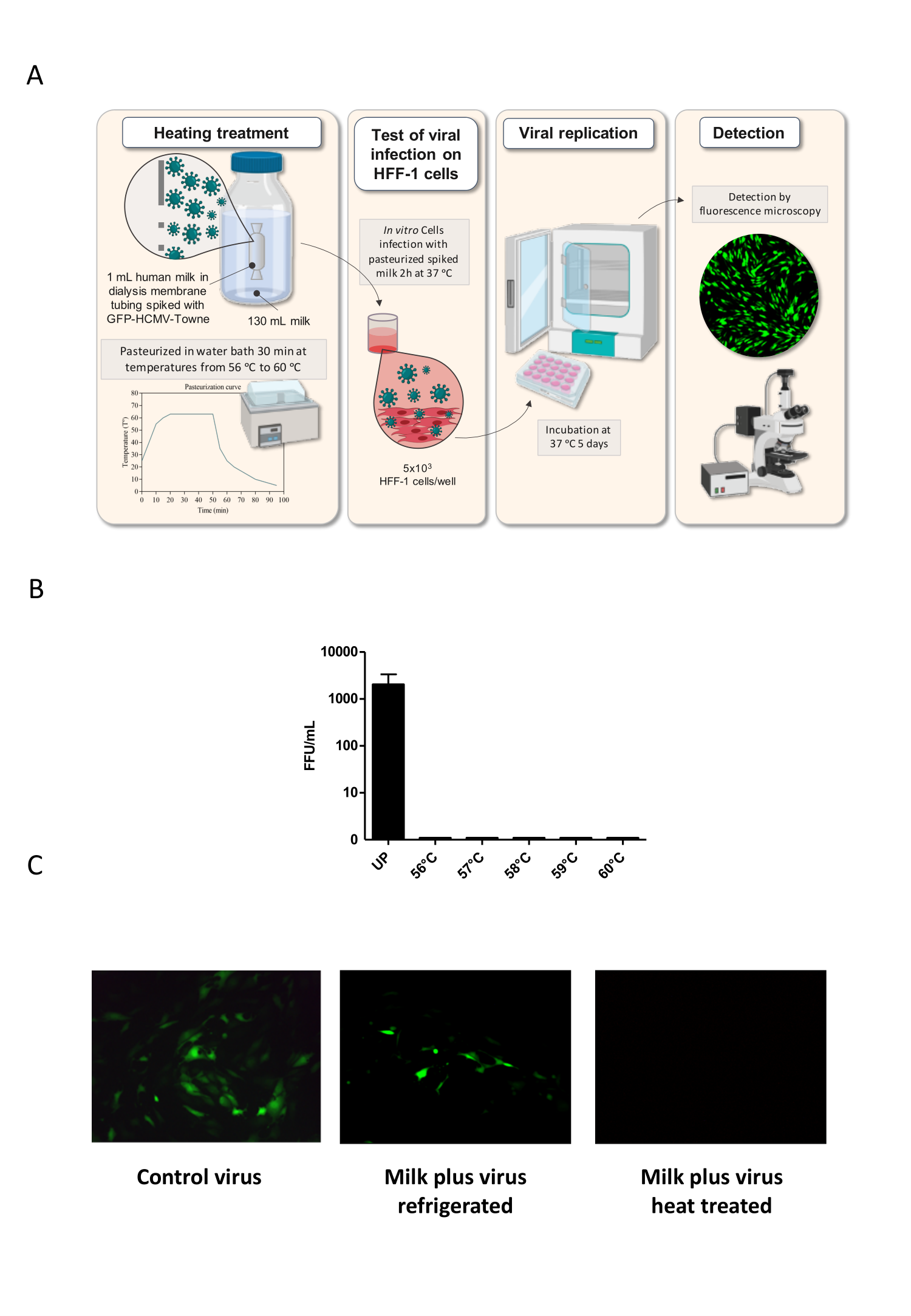
**Supplementary Figure 2.** **Effects of treatment at different temperatures for 30 minutes on HCMV strain Towne infectivity in a pasteurizer-like model.** Panel A. Heat treatment and infection protocol for HCMV. Panel B. In the graph, for each temperature investigated (56-60 °C), HCMV infectivity is reported as foci forming units per mL (FFU/mL). UP, unpasteurised. Panel C. Representative figures of HFF-1 cells treated with HCMV (4 °C for 30 minutes, control virus, left), HCMV-spiked milk samples treated at 4 °C for 30 minutes (control milk plus virus, middle), and at 56 °C for 30 minutes (milk plus virus, right). In green, fluorescent HCMV foci.

**Supplementary Figure 3.** Thermal profiles of pasteurizer-like models of milk spiked with HCMV strain Towne

**56 °C**

**
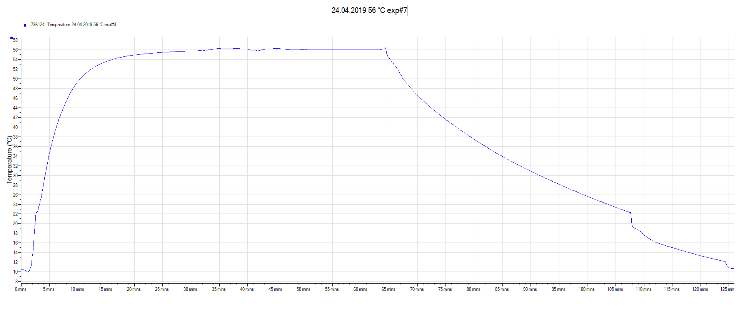
**

**57 °C**

**
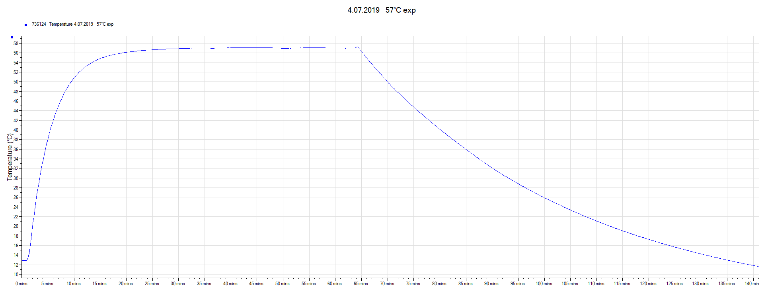
**

**58 °C**

**
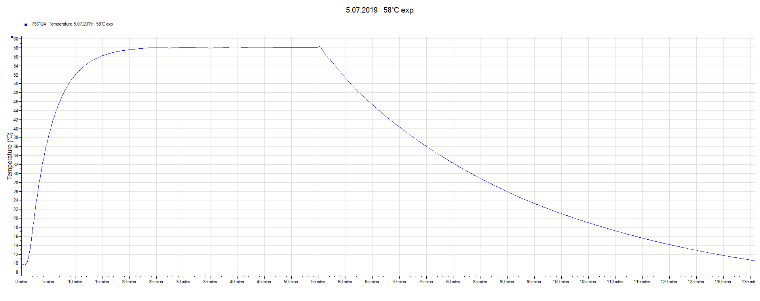
**

**59 °C**

**
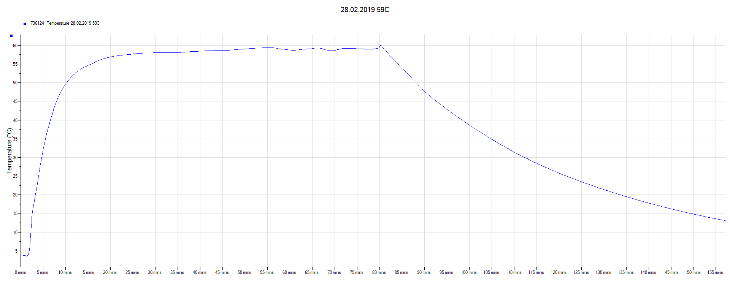
**

**60 °C**

**
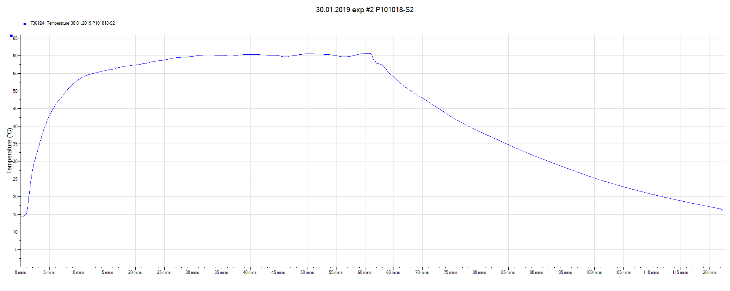
**

**Supplementary Figure 4.** Thermal profile of pasteurizer-like models of milk spiked with HCMV strain AD169.

**60 °C**

**
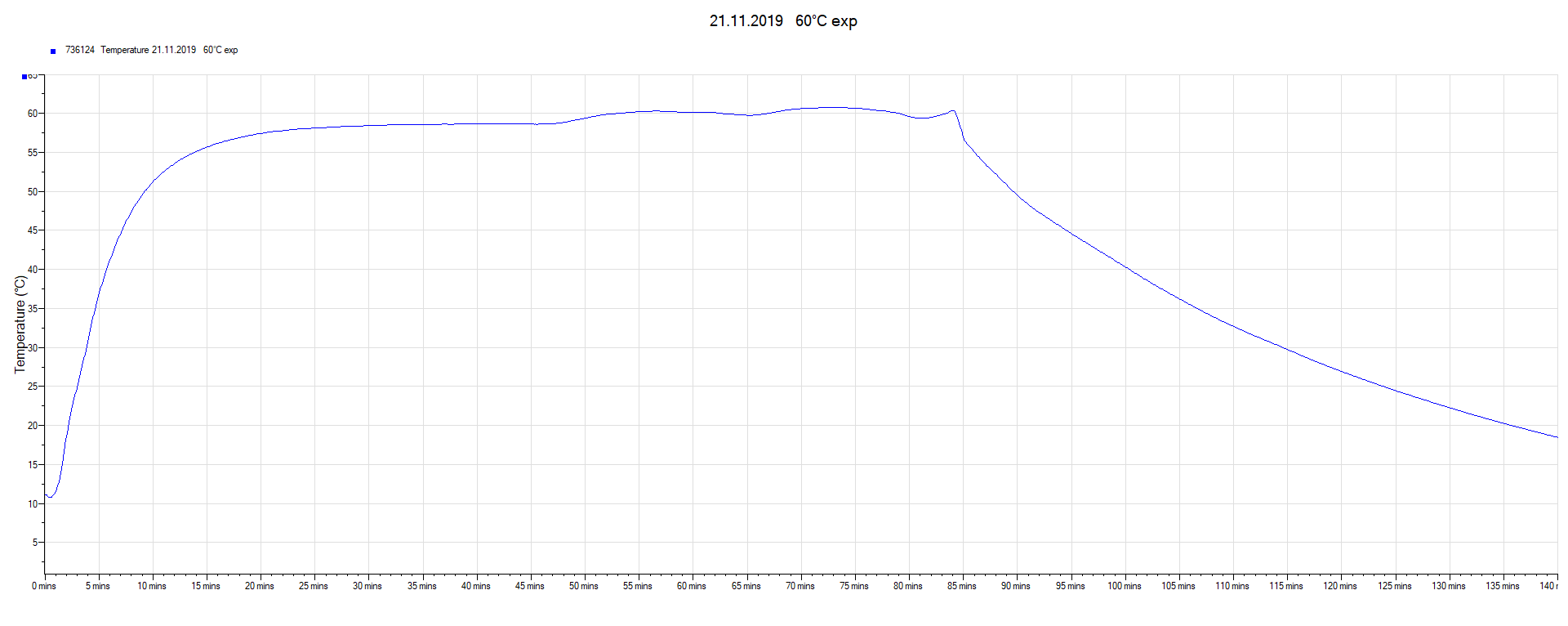
**
